# Highly Radiosensitive Diffuse Large B-cell Lymphoma of the Skull Treated With Low-Dose CyberKnife Radiotherapy: A Case Report

**DOI:** 10.7759/cureus.51227

**Published:** 2023-12-28

**Authors:** Shinichiro Mizumatsu, Ryutaro Nomura

**Affiliations:** 1 Cyberknife Center, Aoyama General Hospital, Toyokawa, JPN; 2 ZAP Center, Kamiyacho Neurosurgical Clinic, Tokyo, JPN

**Keywords:** radiotherapy alone, stereotactic radiosurgery (cyberknife®), abscopal effect, radiosensitive tumor, stereotactic radiotherapy (srt), skull lymphoma, radiosensitivity, low-dose radiotherapy, cyberknife radiotherapy, diffuse large b-cell lymphoma

## Abstract

Diffuse large B-cell lymphoma (DLBCL) of the skull is rare, and there are no reports of treatment using CyberKnife (CK). Here, we report the case of a patient with skull DLBCL treated with low-dose CK radiotherapy (CKR), resulting in effective local control. The patient was a 75-year-old man who was initially diagnosed with multiple skull metastases (frontal, occipital, right orbital bones) from renal pelvic cancer. We initially created a CKR treatment plan for the frontal bone lesion with a marginal dose of 35 Gy and a maximum of 64.8 Gy in five fractions every other day. Because the frontal bone lesion shrank rapidly from the start of the treatment, we completed CKR with a marginal dose of 21 Gy and a maximum of 38.9 Gy in three fractions over five days. At six weeks after CKR, the MRI showed complete resolution of not only the frontal bone lesion but also the occipital and orbital bone lesions that we did not directly target for irradiation. The maximum doses irradiated to the occipital and orbital bone lesions were 0.31 Gy and 0.34 Gy. Because of the marked shrinkage of the skull lesions, we suspected that the patient had a radiosensitive neoplastic disease. FDG-PET/CT revealed multiple lymph nodes and bone metastases. The patient underwent a scrotal biopsy, and the histologic diagnosis was DLBCL. The patient subsequently received chemotherapy for DLBCL. Ten months after CKR and six months after the start of chemotherapy for DLBCL, the patient died due to gastrointestinal bleeding. The skull lesions were well-controlled locally without adverse events due to CKR until the end of the life. Our present case suggests the importance of diagnosis and the effectiveness of low-dose CKR in the skull DLBCL.

## Introduction

Malignant lymphomas (MLs) are generally classified as highly radiosensitive tumors [1]. Radiotherapy alone can cure or result in long-term remission of localized-stage indolent lymphoma [2,3]. Recent studies have reported that ultra-low-dose radiotherapy (LDRT) (2 Gy x 2) is effective for the local palliation of localized-stage indolent lymphoma [4-7]. However, there is a lack of information on the application of LDRT for treating intermediate-grade lymphomas. Diffuse large B-cell lymphoma (DLBCL) is the most common ML type and is classified as an intermediate-grade lymphoma. However, skull DLBCL is rare, and its diagnosis and treatment can be challenging. A large subcutaneous mass with infiltrative bone destruction is the most characteristic feature of cranial vault lymphoma [8]. The optimum treatment for ML of the skull has not been established. In general, surgical resection followed by radio- and chemotherapy has been performed for skull DLBCL. Umemura et al. reported two cases of skull DLBCL [8]. In the first case, it was not possible to administer chemotherapy because of renal failure, but whole-brain radiotherapy (45 Gy) and corticosteroids were administered after surgery. The left parietal tumor disappeared, and the patient was discharged without neurological deficits. In the second case, an occipital craniectomy was performed, with partial tumor excision. Postoperative chemotherapy with high-dose methotrexate was initiated because of the intradural invasion but was not effective. The chemotherapy regimen was changed to rituximab, cyclophosphamide, cytarabine, etoposide, and dexamethasone (the CHASER regimen). The visual disturbance and headache improved rapidly, and the patient was discharged without neurological deficits. Kosugi et al. reported that the skull DLBCL was treated by irradiation (45 Gy) of the mass on the parietal bone and with rituximab, pirarubicin, cyclophosphamide, and vincristine [9]. The patient achieved complete remission after three cycles of systemic chemotherapy. As of 30 months after presentation, no signs of lymphoma have been found. CyberKnife® (Accuray Incorporated, Sunnyvale, CA, USA) is an image-guided stereotactic radiotherapy system that consists of a robotic arm, linear accelerator, and target tracking system [10]. Here, we describe the case of a patient with skull DLBCL that responded markedly to low-dose CyberKnife® radiotherapy (CKR).

## Case presentation

The patient was a 75-year-old man referred to our hospital. He had a medical history of prostate cancer, hypertension, chronic heart failure, chronic kidney disease, and colorectal cancer. At 67 years of age, he underwent a laparoscopic total nephroterectomy for left renal pelvic cancer (RPC). The histology was urothelial carcinoma. Five years and eight months after left kidney surgery, the patient had a recurrence of the right kidney. The patient opted for the best supportive care without aggressive cancer treatment.

Two years and one month after the recurrence, the patient became aware of a frontal lesion. Subsequently, the frontal lesion increased rapidly, and an occipital lesion and numbness in the right lateral orbit also appeared. The patient was diagnosed with skull metastasis from RPC at the urology department of the previous hospital. One month after the onset of the frontal bone lesion, the patient visited our hospital for CKR.

**Figure 1:**
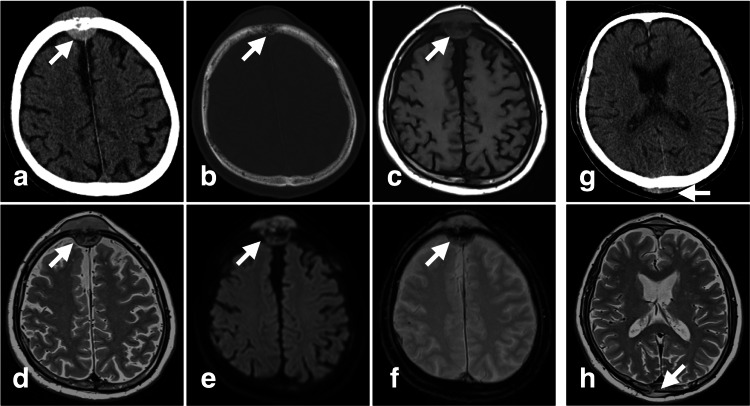
CT and MRI before CKR The frontal bone lesion (white arrows): (a) plain CT, (b) bone CT, (c) T1WI, (d) T2WI, (e) DWI, (f) T2*. The occipital bone lesion (white arrows): (g) plain CT, (h) T2WI CT: computed tomography, MRI: magnetic resonance imaging, CKR: CyberKnife radiotherapy, bone-CT: bone window computed tomography, T1WI: T1-weighted image, T2WI: T2-weighted image, DWI: diffusion-weighted image

**Figure 2:**
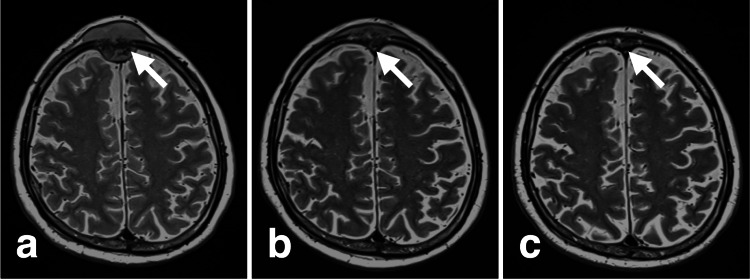
Time course of the frontal lesion on MRI T2WI (white arrows) (a) pre-CKR, (b) Day 5 after starting CKR, (c) Day 44 after starting CKR MRI: magnetic resonance imaging, T2WI: T2-weighted image, CKR: CyberKnife radiotherapy

At the first visit, the patient presented with increasing frontal and occipital masses, right lateral eyelid swelling, and numbness in the right temporal region of the face. Computed tomography (CT) and magnetic resonance imaging (MRI) showed an invasive lesion growing inside and outside the frontal bone (Figures 1a-1f, 2a).

**Figure 3:**
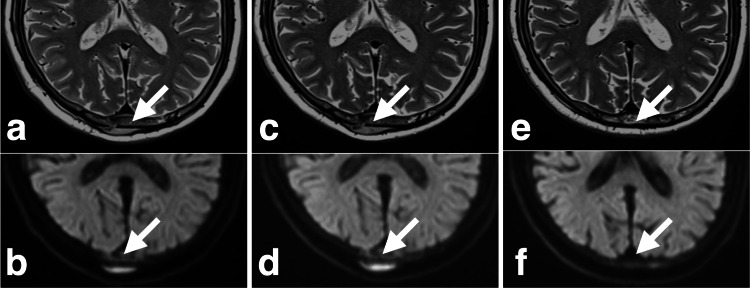
Time course of the occipital lesion on MRI T2WI and DWI (white arrows) pre-CKR: (a) T2WI, (b) DWI. Day 5 after starting CKR: (c) T2WI, (d) DWI. Day 44 after starting CKR: (e) T2WI, (f) DWI. MRI: magnetic resonance imaging, T2WI: T2-weighted image, DWI: diffusion-weighted image, CKR: CyberKnife radiotherapy

**Figure 4:**
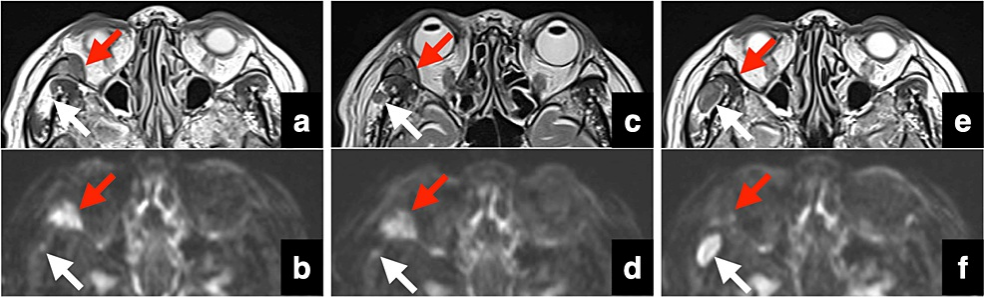
Time course of the right orbital lesion (red arrows) and temporal fossa (white arrow) before and after CKR on MRI T2WI and DWI pre-CKR: (a) T2WI, (b) DWI. Day 5 after starting CKR: (c) T2WI, (d) DWI. Day 44 after starting CKR: (e) T2WI, (f) DWI CKR: CyberKnife radiotherapy, MRI: magnetic resonance imaging, T2WI: T2-weighted image, DWI: diffusion-weighted image

Lesions were also observed in the occipital and right orbital bone (Figures 1g, 1h, 3a, 3b, 4a, 4b).

**Figure 5:**
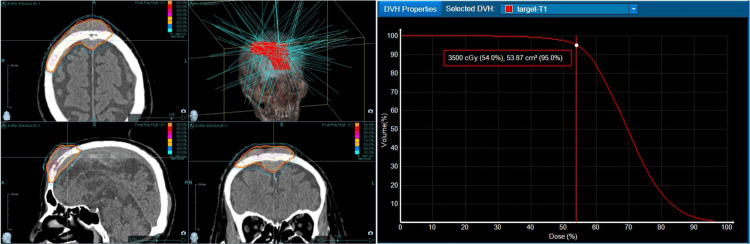
CKR plan using MultiPlan for the frontal bone lesion There are the dose distributions and DVH in MultiPlan® (Accuray Incorporated, Sunnyvale, CA, USA). CKR: CyberKnife radiotherapy, DVH: dose-volume histogram, PTV: planning target volume

We decided to treat the frontal bone lesion first because it was bulky in size and had rapid growth. We created an initial CKR treatment plan with a marginal dose (the minimum dose to 95 % of the planning target volume (D95)) of 35 Gy and a maximum of 64.8 Gy to be delivered in five fractions every alternate day (Figure 5).

The first CKR (day 1) was performed without any issues. On day 3 (the second CKR), we observed that the frontal lesion had already shrunk. The swelling and numbness due to skull lesions had improved by day 3. On day 5 (the third CKR), the frontal lesion had further reduced size. On the same day, an MRI showed that the frontal bone lesion had significantly shrunk (Figure 2b). Therefore, we stopped the remaining CKR until the third CKR. A new lesion had appeared in the right temporal fossa on the dorsal side of the right orbital bone lesion on MRI (Figures 4c, 4d). The size of the occipital and orbital bone lesions did not differ considerably on CKR day 5 MRI (Figures 3c, 3d, 4c, 4d). Finally, the actual CKR performed on the frontal bone lesion was a marginal dose (D95) of 21 Gy, with a maximum of 39 Gy, divided into three fractions over five days (Table 1).

**Table 1:** CKR treatment parameters of actual irradiated dose to the lesions The total treatment was over five days. There was no marginal expansion in all PTV except the frontal bone lesion. The prescribed dose was D95. CKR: CyberKnife radiotherapy, D95: the minimum dose to 95% of the PTV, PTV: planning target volume, CR: complete response

	Frontal bone	Occipital bone	Orbital bone	Temporal fossa
PTV (mL)	56.72	5.31	6.03	no lesion
Prescription dose (Gy)	21	0.15	0.12	no lesion
Maximum dose (Gy)	39	0.31	0.34	0.25
Result	CR	CR	CR	new lesion

The maximum doses administered to the surrounding critical organs were 18.22 Gy to the skin surface, 0.52 Gy to the right eye, 0.62 Gy to the left eye, 2.18 Gy to the right optic nerve, and 0.71 Gy to the left optic nerve.

Two weeks after CKR, the patient underwent the first chemotherapy (gemcitabine and carboplatin) for treating RPC. Dexamethasone (DEX) (8.2 mg) was used together with the chemotherapy. The chemotherapy was discontinued only after one session due to neutropenia. Six weeks after CKR, a follow-up MRI showed complete resolution of the frontal lesion (Figure 2c). The occipital and right orbital bone lesions, which were not the direct targets of CKR, had also completely disappeared on the follow-up MRI (Figures 3e, 3f, 4e, 4f). The new lesion had grown in the right temporal fossa. The patient was aware that the occipital lesion had shrunk before the start of the chemotherapy.

**Figure 6:**
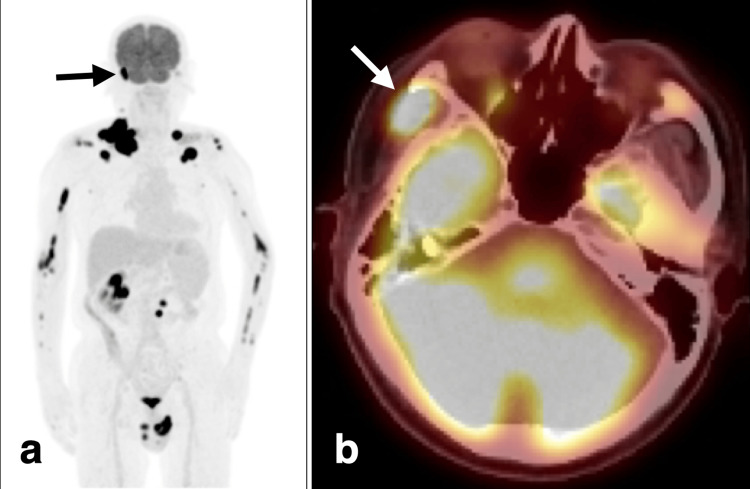
FDG-PET/CT two months after CKR (a) MIP. Multiple lesions with high FDG uptake in the whole body MIP. There was high FDG uptake of the lesion in the right temporal fossa on PET/CT (black arrow). (b) The right temporal lesion with high FDG in the PET/CT (white arrow). FDG: 2-deoxy-2-[18F] fluoro-D-glucose, PET: positron emission tomography, CT: computed tomography, CKR: CyberKnife radiotherapy, MIP: maximum intensity projection

Two months after CKR, the patient underwent 18F-fluorodeoxyglucose (FDG)-positron emission tomography/computed tomography (PET/CT). We suspected that the patient had a hematological malignancy because the examination revealed multiple lymph nodes and bone metastases (Figure 6a).

The new lesion in the right temporal fossa also showed a high uptake of FDG on PET/CT (Figure 6b). There were no FDG uptakes in the frontal, occipital, and right orbital bone lesions. A scrotal biopsy revealed that the pathological diagnosis was DLBCL, stage IV (Lugano classification).

The patient successively received chemotherapy with R (rituximab)-CHOP (cyclophosphamide, doxorubicin, vincristine, and prednisone) for DLBCL. Nine months after CKR and six months after the start of the chemotherapy, the patient died due to gastrointestinal bleeding. The skull lesions were well-controlled locally without any adverse events due to CKR until the end of the life.

## Discussion

ML in the skull is a rare incidence [11]. Nitta et al. reported a detailed review of primary ML in the cranial vault [11]. The frequency of ML occurrence in the parietal, frontal, occipital, and temporal bones was 57%, 54%, 21%, and 15%, respectively. The common symptoms were a growing subcutaneous mass on the scalp (84%), headache (33%), focal neurological deficit (25%), and seizure (6%). The subcutaneous scalp mass was firm (57%), soft (16%), non-tender (56%), tender (22%), or accompanied by reddish skin or ulcer (7%). In patients with a growing mass, the mean duration of growth was 5.9 ± 6.3 months before the patient presented for treatment. The most frequent lymphocyte type was B-cell lymphoma (94%), followed by T-cell lymphoma (6%), and DLBCL was the most common lymphoma. The prognosis was relatively good, with six-month and one-year survival rates of 90.5 % and 83.3%, respectively. Skull ML is characterized by a rapidly growing subcutaneous scalp mass, incomplete bone destruction relative to the expanding subcutaneous lesion, and poor vascularization. In the present case, we should have also considered skull ML before CKR because of the rapidly enlarging subcutaneous lesions and mild bone destruction. It remains unknown whether the primary lesion was the skull because FDG-PET/CT showed multiple lesions throughout the body.

ML is highly radiosensitive regardless of histology [1]. The usefulness of LDRT for slowly progressive ML has been reported [2-7]. However, few studies have reported the application of low-dose radiation therapy for treating intermediate-grade lymphomas such as DLBCL. Recently, the usefulness of LDRT for treating DLBCL has been reported in several cases [12-16].

Murthy et al. reported that LDRT (2Gy x 2) was performed on the DLBCL group and the non-DLBCL group, and the effect of the DLBCL group (overall response rate [ORR] of 50 % and no complete remission [CR]) was inferior to that of the non-DLBCL group (ORR: 86%; CR: 40%) [12]. Matoba et al. reported good results with single irradiation radiotherapy using three-dimensional conformal radiotherapy for DLBCL in the nasopharynx that recurred after radiochemotherapy (chemotherapy: CHOP; radiotherapy: 23 fractions, total dose of 46 Gy) and was resistant to additional chemotherapy (R-CHOP, irinotecan, and dexamethasone) [13]. After the radiotherapy, they found the lesion shrinkage and symptom relief several days later, and CR was observed on the CT one month later. There was no severe acute toxicity related to the radiotherapy. There was no local recurrence until death due to recurrence outside the radiation field four months after radiotherapy. Tanaka et al. recommended radiotherapy with a single-fraction 8 Gy dose after using a single-fraction LDRT (2, 4, and 8 Gy) for six patients with DLBCL after systemic chemotherapy [14]. Furlan et al. reported that LDRT (2 x 2 Gy) is effective for palliation in patients with DLBCL, based on the results of a phase II trial in which the overall response rate was 70% (16 of 23 patients), with seven complete responses and nine partial responses (median duration of response, six months; range, 1-39 months) [15]. McNeil et al. reported palliative radiotherapy for necrotic right upper neck DLBCL [16]. They delivered the lesion at a dose of 25 Gy in five fractions on alternate days over two consecutive weeks. One month later, the lesion had a complete response without residual symptoms.

In the present case, the CKR delivered to the frontal bone DLBCL was 21 Gy in 3 fractions on alternate days for 5 consecutive days. The occipital and orbital lesions, which were not directly targeted by the CKR, were irradiated with a maximum dose of 0.31 and 0.34 Gy, respectively (Table 1). The initial CKR plan was with five fractions. Because the frontal bone lesion had significantly shrunk until Day 5, we stopped the remaining CKR after the third CKR. A lower dose might be sufficient to affect the skull DLBCL because the lesion shrinkage was remarkable after the first or the second CKR. We also considered the possibility that the chemotherapy and DEX affected the occipital and orbital bone lesions. However, we determined that the main effect was the radiotherapy because the patient already noticed a significant shrinkage of the occipital bone lesion even before the chemotherapy. Because the irradiated dose was extremely low, we hypothesized that the shrinkage of the occipital and orbital bone lesions may have been due not only to the direct radiotherapy effect but also to other effects, such as the abscopal effect [17,18]. The right temporal fossa area of the new lesion appearance was irradiated with a maximum dose of 0.25 Gy (Table 1). However, the growth of the new lesion was not suppressed. The useful lower dose limit of radiotherapy for DLBCL is unknown because of the small number of reported cases.

Stereotactic radiotherapy (SRT) is a novel technique that allows more radiation to reach the lesion while reducing the amount of surrounding normal tissue irradiated. CK is an image-guided SRT system with a robotic arm, linear accelerator, and target-tracking system [10]. The advantages of CKR are that dose reduction to organs at risk such as the eye, brain, and skin can be easily achieved and that early therapeutic effects can be expected in large lesions using a high intratumoral dose. In general, LDRT has the advantage of fewer adverse events. Moreover, in our present case, the patient showed a rapid response to treatment by the CKR.

The skull DLBCL has the problem of the small number of reported cases and the difficulty in conducting prospective studies. Therefore, the treatment strategy and details of the skull DLBCL radiotherapy have not been determined. With the recent advances in radiotherapy technology, it is essential to record more case reports in the future to develop optimal therapeutic strategies for skull DLBCL.

## Conclusions

Skull DLBCL is rare, and there were only a few reports on this subject. Here, we described the case of a patient with skull DLBCL, which was initially diagnosed with metastases from RPC and successfully treated with low-dose CKR. The present case suggested the importance of diagnosis and the usefulness of low-dose CKR for treating skull DLBCL. Tumor radiosensitivity is an essential factor in radiotherapy, and we should reconsider the treatment plan if the results differ from those expected. CKR has the advantages of fewer adverse events and faster treatment effects than CRT. Low-dose CKR for DLBCL may be an effective local treatment option. However, the details of the radiotherapy for the skull DLBCL, such as the optimal dose and fractions, have not been determined. It is necessary to accumulate additional clinical data on whether low-dose CK is an effective treatment option for the skull DLBCL.
